# Crystal structure analysis of the biologically active drug mol­ecule riluzole and riluzolium chloride

**DOI:** 10.1107/S2056989019009022

**Published:** 2019-07-02

**Authors:** Pradip Kumar Mondal, Athulbabu T, Varun Rao, Deepak Chopra

**Affiliations:** a Department of Chemistry, Indian Institute of Science Education and Research, Bhopal, Bhauri, Bhopal 462066, India

**Keywords:** crystal structure, riluzole, mol­ecular salt, weak inter­actions, electrostatic potential

## Abstract

This study is an investigation into the crystal structure of the biologically active drug mol­ecule riluzole [RZ, 6-(tri­fluoro­meth­oxy)-1,3-benzo­thia­zol-2-amine], C_8_H_5_F_3_N_2_OS, and its derivative, the riluzolium chloride salt [RZHCl, 2-amino-6-(tri­fluoro­meth­oxy)-1,3-benzo­thia­zol-3-ium chloride], C_8_H_6_F_3_N_2_OS^+^·Cl^−^. In spite of repeated efforts to crystallize the drug, its crystal structure has not been reported to date, hence the current study provides a method for obtaining crystals of both riluzole and its corresponding salt, riluzolium hydro­chloride. The salt was obtained by grinding HCl with the drug and crystallizing the obtained solid from di­chloro­methane. The crystals of riluzole were obtained in the presence of l-glutamic acid and d-glutamic acid in separate experiments. In the crystal structure of RZHCl, the –OCF_3_ moiety is perpendicular to the mol­ecular plane containing the riluzolium ion, as can be seen by the torsion angle of 107.4 (3)°. In the case of riluzole, the torsion angles of the four different mol­ecules in the asymmetric unit show that in three cases the tri­fluoro­meth­oxy group is perpendicular to the riluzole mol­ecular plane and only in one mol­ecule does the –OCF_3_ group lie in the same mol­ecular plane. The crystal structure of riluzole primarily consists of strong N—H⋯N hydrogen bonds along with weak C—H⋯F, C—H⋯S, F⋯F, C⋯C and C⋯S inter­actions, while that of its salt is stabilized by strong [N—H]^+^⋯Cl^−^ and weak C—H⋯Cl^−^, N—H⋯S, C—H⋯F, C⋯C, S⋯N and S⋯Cl^−^ inter­actions.

## Abstract 2

An investigation into the crystallization, crystal structure and packing analysis of the biologically active drug mol­ecule riluzole and its derivative, the riluzolium chloride salt, has been carried out.

## Chemical Context   

Crystals are composed of an infinite array of atoms or mol­ecules arranged in a regular pattern in space. Such crystals form assemblies of supra­molecules (Desiraju, 2013 ▸; Yan & Huang, 2010 ▸). These supra­molecular assemblies are formed by the involvement of certain inter­molecular inter­actions (Mondal, Kiran *et al.*, 2017 ▸). The study of these inter­molecular inter­actions is significant in both chemistry (Raynal *et al.*, 2014 ▸) and biology (Ball & Maechling, 2009 ▸). Some of the major inter­molecular inter­actions are hydrogen-bonding, dipole–dipole, van der Waals and halogen inter­actions (Paulini *et al.*, 2005 ▸). Understanding the essential mol­ecular inter­actions and synthons involved in the early stages of nucleation is very important in determining the formation of crystals (Davey *et al.*, 2013 ▸). These packing trends and supra­molecular synthons can also repeat themselves in other crystal structures with similar functional groups. The phenomenon of polymorphism is also a common occurrence because of the possible presence of diverse combinations of inter­molecular inter­actions (Cruz-Cabeza & Bernstein, 2014 ▸).

Riluzole (RZ) is the only available drug used for the treatment of amyotrophic lateral sclerosis (ALS) and diseases like Parkinson’s disease, Huntington’s disease and other mood and anxiety disorders (Nakane *et al.*, 2016 ▸). Even though riluzole is a most important pharmaceutical drug (Doble, 1996 ▸), no crystal structure of pure riluzole has been obtained to date, although several methods have been tried in the past (Mondal, Rao, *et al.*, 2017 ▸; Mondal *et al.*, 2018 ▸; Thomas *et al.*, 2019 ▸; Yadav *et al.*, 2018 ▸).
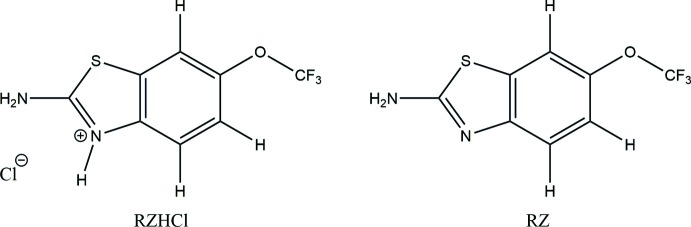


In this work, we have been successful in obtaining crystals of riluzole along with those of its hydro­chloride salt. An in-depth analysis of the two crystal structures has been performed and the role of strong hydrogen bonds and weak inter­molecular inter­actions in the crystal lattice has been established.

## Structural commentary   

**Figure 1:**
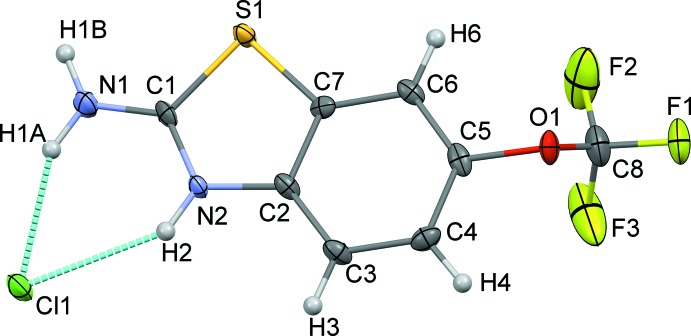
*ORTEP* view of riluzolium chloride drawn with 50% ellipsoidal probability. The dotted lines depict inter­molecular inter­actions in the asymmetric unit.

**Figure 2:**
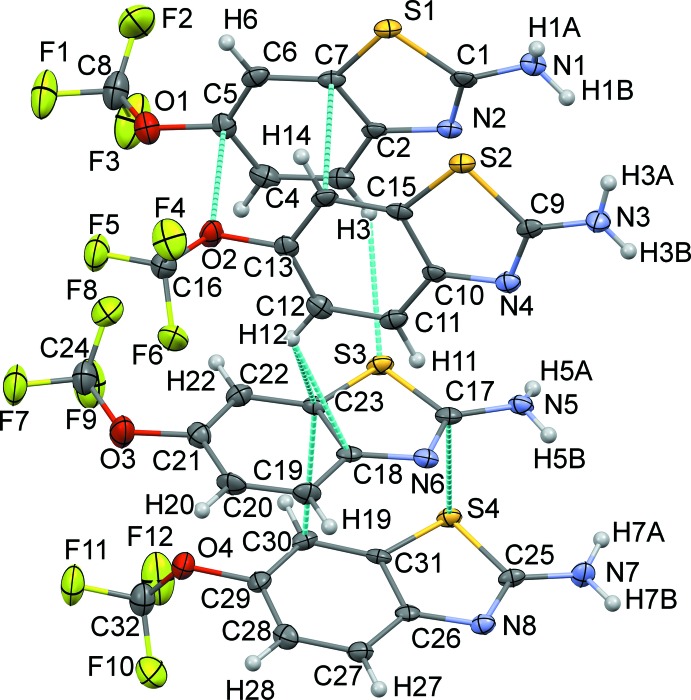
*ORTEP* view of riluzole drawn with 50% ellipsoidal probability. The dotted lines depict inter­molecular inter­actions in the asymmetric unit.

The riluzolium chloride salt crystallizes in the *P*2_1_/*c* space group with one riluzolium cation (RZH^+^) and a chloride anion (Cl^−^) in the asymmetric unit while the riluzole mol­ecule crystallizes in the centrosymmetric triclinic *P* 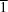 space group with *Z*′ = 4. The asymmetric unit of riluzolium chloride (Fig. 1 ▸) shows a riluzolium ion with a chloride ion held *via* [N—H]^+^⋯Cl^−^ inter­actions between the riluzolium cation and the chloride anion. On the other hand, the asymmetric unit of riluzole (Fig. 2 ▸) comprises four mol­ecules, wherein each pair is perpendicular to the other pair, with parallel pairs being held together by C⋯C, C⋯O and C⋯S inter­molecular contacts and each pair is connected with the other pair *via* C—H⋯π or C—H⋯S hydrogen-bonding inter­actions. The conformations of riluzole and of the riluzolium cation in the crystal packing are preserved except for the conformational changes that occur in the –OCF_3_ group. The main difference between the two mol­ecular structures can be seen from the magnitude of the torsion angles C*i*—C*j*—O*k*—C*l*, Table 1 ▸ (Mondal, Rao *et al.*, 2017 ▸; Mondal *et al.*, 2018 ▸; Thomas *et al.*, 2019 ▸; Yadav *et al.*, 2018 ▸). Both the structures in the current study crystallized in a centrosymmetric space group. Hence, only torsion angles within the 0 to 180° range are significant. In the crystal structure of RZHCl, the torsion angle relative to the –OCF_3_ moiety is 107.4 (3)°, which means that the tri­fluoro­meth­oxy group is roughly perpendicular to the mol­ecular plane of the riluzolium ion. The corresponding torsion angles for the four different riluzole mol­ecules in the asymmetric unit of the crystal structure of RZ are −86.2 (4), 91.9 (3), −96.4 (3)° (when the –OCF_3_ group is perpendicular to the mol­ecular plane of riluzole) and 167.6 (2)° (for one mol­ecule when the group is in the same mol­ecular plane).

## Supra­molecular features   

**Figure 3:**
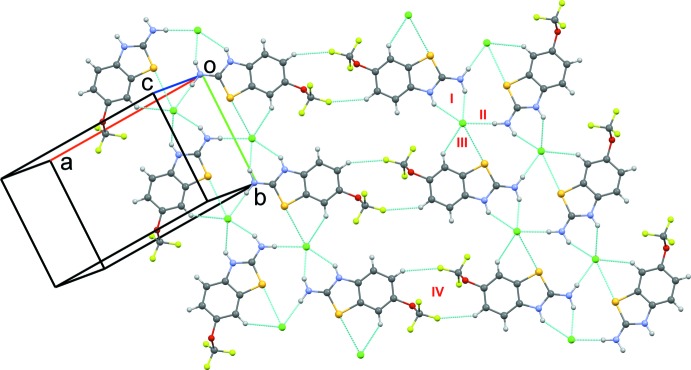
A comparative view of the packing of riluzolium chloride represented *via* N—H⋯Cl, C—H⋯Cl, C—H⋯F, and S⋯Cl inter­molecular inter­actions. Dotted pale-blue lines depict the inter­molecular inter­actions.

**Figure 4:**
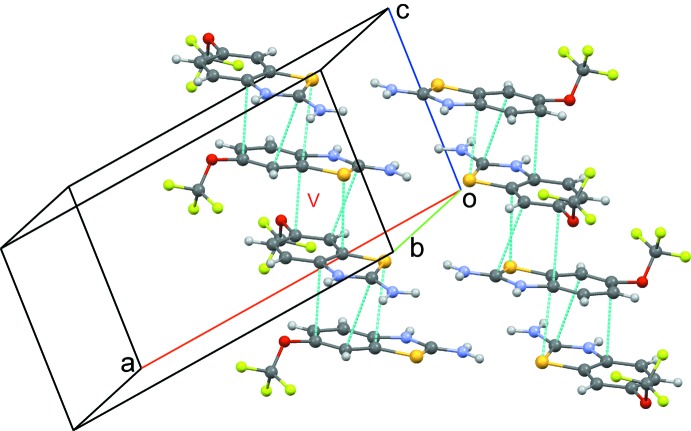
A comparative view of the packing of riluzolium chloride represented *via* C⋯C and C⋯S inter­molecular inter­actions. Dotted pale-blue lines depict the inter­molecular inter­actions.

**Figure 5:**
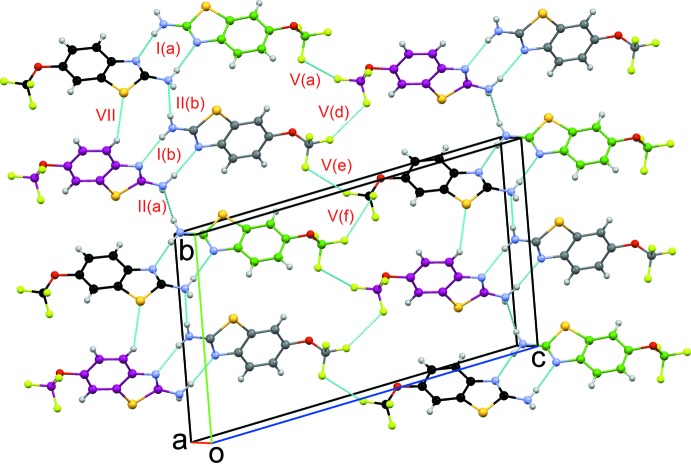
Packing of mol­ecules with strong N—H⋯N dimers formed along the *bc* plane with weak C—H⋯S and F⋯F inter­actions in riluzole. Dotted lines depict the inter­molecular inter­actions, and different colours for C atoms have been used for *Z*′ > 1.

**Figure 6:**
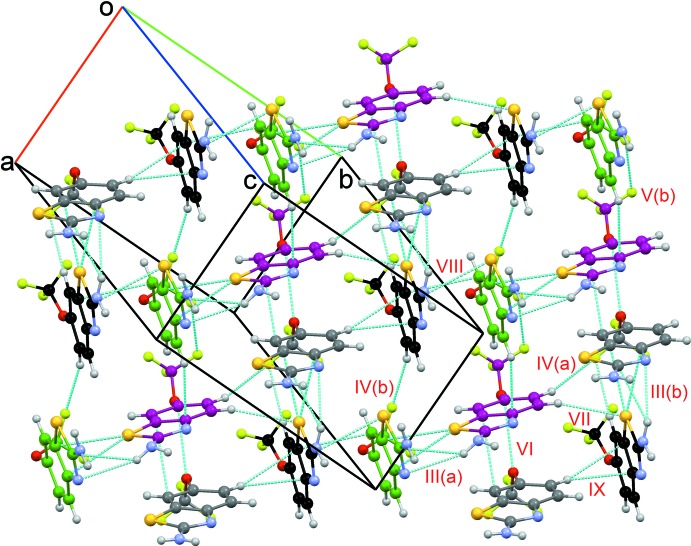
Packing of mol­ecules with weak C—H⋯F, C—H⋯S, F⋯F, C⋯C, C⋯O, C—H⋯C and C⋯S inter­actions in riluzole. Dotted lines depict the inter­molecular inter­actions, and different colours for C atoms have been used for *Z*′ > 1.

The riluzolium ion forms hydrogen-bonding inter­actions (Table 2 ▸) with a chloride ion *via* strong N1—H1*A*⋯Cl1 (2.15 Å, 154°), N2—H2⋯Cl1 (2.35 Å, 139°) and N1—H1*B*⋯Cl1 (2.14 Å, 175°) inter­actions (Motifs I and II, Fig. 3 ▸) along with weak C—H⋯Cl and S⋯Cl inter­actions (Motif III), forming a mol­ecular sheet down the *ab* plane. Riluzolinium mol­ecules in parallel planes are connected by weak C⋯C and C⋯S inter­actions (Motif V, Fig. 4 ▸). Two such chains along the *b* axis are connected *via* motif IV, the dimer based on two symmetry-related C—H⋯F–C*sp* ^3^ inter­actions, which yields an 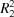
(12) graph-set motif. The importance of such inter­actions has been evidenced in the crystal structures of –F- and –CF_3_-containing benzanilides (Panini *et al.*, 2016 ▸). The crystal structure of riluzole consists of strong as well as weak inter­actions between the corresponding riluzole mol­ecules. Similar types of inter­actions are grouped together as motifs, in both parallel and perpendicularly aligned mol­ecules in the asymmetric unit. Strong N—H⋯N hydrogen-bonded 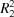
(8) dimers are obtained (Motifs I to III; Figs. 5 ▸, 6 ▸), leading to the formation of chains along the *b*-axis direction. [Motifs I(*a*) and I(*b*); Fig. 5 ▸]. In addition, the amine nitro­gen forms hydrogen-bonding inter­actions with the amine hydrogen of another riluzole mol­ecule [Motifs II(*a*) and II(*b*); Fig. 5 ▸]. The ring nitro­gen atom was found to form hydrogen bonds with the amine hydrogens [Motifs III(*a*) and III(*b*)] along with other weak C—H⋯F, N—H⋯C, and C⋯S inter­actions. Mol­ecular motifs IV(*a*), IV(*b*), and V(*a*–*f*), show the presence of short and highly directional inter­actions involving organic fluorine, such as the C*sp* ^3^—F⋯H–C*sp* ^2^ (2.46 Å, 161°; 2.41 Å, 161°) hydrogen bond and the C*sp* ^3^—F⋯F—C*sp* ^3^ (2.907 Å, 137°, 107°; 2.923 Å, 115°, 120°; 2.845 Å, 127°, 127°) inter­actions [Figs. 5 ▸ and 6 ▸], in the crystal packing and these structural features are indeed noteworthy. Furthermore, we have also observed sulfur forming weak C—H⋯S and C⋯S inter­actions (Motifs VII and VIII) in addition to the presence of weak C⋯O, C⋯C (Motif VI), and C—H⋯C inter­actions (Motif IX) (Fig. 6 ▸).

**Figure 7:**
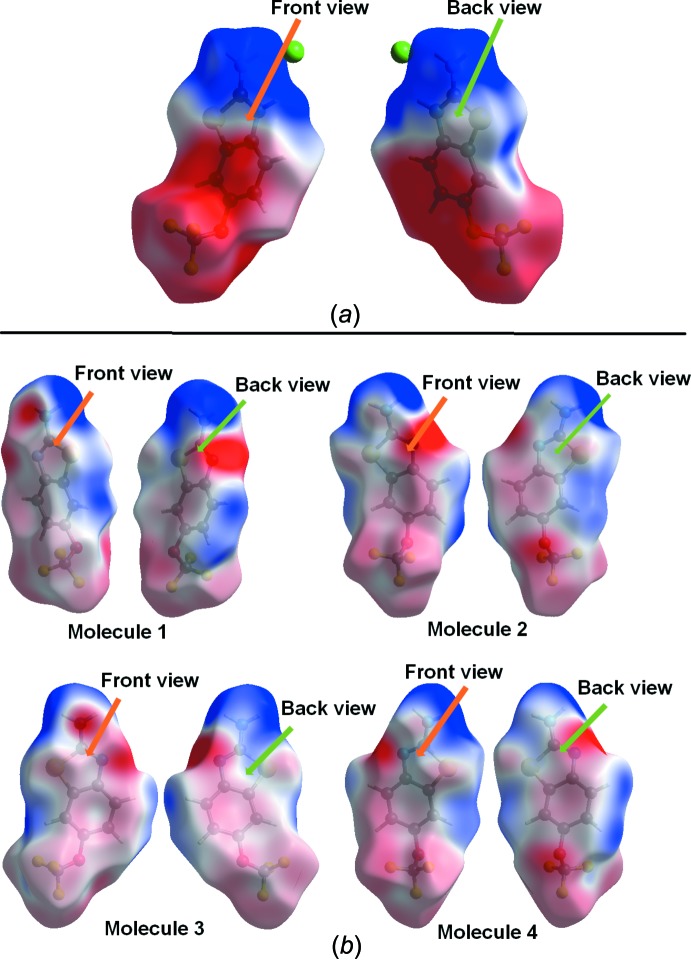
Electrostatic potential (ESP) mapped on the Hirshfeld surfaces of (*a*) the RZHCl salt and (*b*) RZ (four mol­ecules), over the range −0.05 au (red) through 0.0 (white) to 0.05 au (blue).

The electrostatic potentials (ESP) (Spackman *et al.*, 2008 ▸) were mapped on the Hirshfeld surfaces for RZHCl (Fig. 7 ▸ *a*), and for the four mol­ecules in RZ (Fig. 7 ▸ *b*, front and back views). These were calculated using HF/6-31G** *ab initio* wave functions *via* the program *Gaussian09* (Frisch *et al.*, 2009 ▸). The ESP map allows a qu­anti­tative understanding of the nature of electron-rich and electron-deficient sites in the mol­ecule to be obtained. As expected in all the RZ mol­ecules, the electronegative regions are around the nitro­gen, oxygen, fluorine, and sulfur atoms. The corresponding electropositive regions were observed around the N—H and C—H bonds.

## Database analysis   

Recently, Thomas and coworkers (Thomas *et al.*, 2019 ▸) reported the ubiquity of a robust, directional S⋯O chalcogen-bonded synthon and have probed the electronic nature in a series of co-crystals and salts of the drug riluzole. The S⋯O bond order for chalcogen bonding was found to be one-third of a single bond (minimum 0.10 to maximum 0.35), and these are short (2.90 to 3.40 Å) and directional (<C—S⋯O = 160–179°) in nature. In another recent study, performed on the drug riluzole, the riluzole mol­ecules (CCDC codes YEPJIP and YEPJOV; Yadav *et al.*, 2018 ▸) also display the presence of S⋯O chalcogen-bonded synthons (S⋯O distances = 3.39 and 3.42 Å, respectively). However, in the current study, S⋯O chalcogen-bonded synthons were not observed.

## Synthesis and crystallization   

Riluzole was obtained from Rallis India Ltd, and different solvents were used to crystallize it, along with two additives, namely l-Glutamic acid (LGA) and d-Glutamic acid (DGA), which were obtained from Sigma Aldrich and used directly without further purification. The crystallization of riluzole was conducted with LGA and DGA, by the solvent-drop grinding method. Grinding was carried out for 15-20 minutes, with the dropwise addition of methanol at an inter­val of 5 min in an agate mortar and pestle. The slow evaporation method was conducted both at low temperature (278 K) in a refrigerator and also at room temperature with 5 mg of granulated material for each crystallization. This resulted in the formation of plate-like crystals of riluzole from methanol. The riluzole crystals were collected from the crystallization beaker under the polarizing microscope and used for single crystal XRD experiments. No further experiments to evaluate the role of additives have been performed and these are not within the scope of the current work.

Riluzolium chloride was obtained by grinding concentrated HCl (35%) with riluzole in a 1:1 molar ratio for 10-15 minutes and the powder obtained was recrystallized from different solvents. 5 mg of granulated material was used for each crystallization. In particular, crystals of riluzolium chloride were obtained from di­chloro­methane (DCM).

## Refinement   

Crystal data, data collection and structure refinement details are summarized in Table 3 ▸. All hydrogen atoms attached to the carbon atoms and *sp* ^2^ nitro­gen atoms were placed in calculated positions (C–H = 0.95 Å and N*sp* ^2^—H = 0.88 Å) and refined as riding with *U*_iso_(H) = 1.2*U*_eq_(C, N*sp* ^2^). Hydrogen atoms attached to *sp* ^3^ nitro­gen atoms were located in difference-Fourier maps (N*sp* ^3^—H = 0.81–0.91 Å). The normalized values of hydrogen atoms given by *PARST* (Nardelli, 1995 ▸) were used for the hydrogen-bonding (Taylor & Kennard, 1983 ▸) analysis.

## Supplementary Material

Crystal structure: contains datablock(s) RZHCl, RZ. DOI: 10.1107/S2056989019009022/xi2017sup1.cif

Structure factors: contains datablock(s) RZHCl. DOI: 10.1107/S2056989019009022/xi2017RZHClsup2.hkl

Structure factors: contains datablock(s) RZ. DOI: 10.1107/S2056989019009022/xi2017RZsup3.hkl

Supporting information file. DOI: 10.1107/S2056989019009022/xi2017RZHClsup4.cml

Supporting information file. DOI: 10.1107/S2056989019009022/xi2017RZsup5.cml

CCDC references: 1820861, 1820860

Additional supporting information:  crystallographic information; 3D view; checkCIF report

## Figures and Tables

**Table 1:** List of torsion angles (°)

Compound	C*i*—C*j*—O*k*—C*l*	Torsion
RZHCl	C4—C5—O1—C8	107.4 (3)
RZ	C4—C5—O1—C8	−86.2 (4)
	C12—C13—O2—C16	91.9 (3)
	C20—C21—O3—C24	167.6 (2)
	C28—C29—O4—C32	−96.4 (3)

**Table 2:** Inter­molecular inter­actions (Å, °) in the crystal structure of the RZHCl salt and RZ

Motif number	Symmetry Code	Possible involved inter­actions	Geometry
RZHCL			
I	*x*, *y*, *z*	N1—H1*A*⋯Cl1	2.15, 154
		N2—H2⋯Cl1	2.35, 139
II	−*x*, 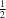 + *y*, 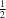 − *z*	N1—H1*B*⋯Cl1	2.14, 175
III	*x*, 1 + *y*, *z*	C6—H6⋯Cl1	2.60, 135
		S1⋯Cl1	3.340 (2)
IV	1 − *x*, 2 − *y*, 2 − *z*	C4—H4⋯F1	2.57, 147
V	*x*, 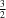 − *y*, 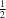 + *z*	C5⋯C2	3.289 (7)
		C6⋯C1	3.292 (7)
		C7⋯S1	3.456 (6)
			
RZ			
I(*a*)	−*x*, 2 − *y*, 2 − *z*	N7—H7*B*⋯N6	1.89, 170
		N5—H5*B*⋯N8	2.03, 175
I(*b*)	1 − *x*, 1 − *y*, 2 − *z*	N3—H3*B*⋯N2	1.92, 167
		N1—H1*B*⋯N4	2.06, 170
II(*a*)	−*x*, 1 − *y*, 2 − *z*	N7—H7*A*⋯N1	2.14, 169
II(*b*)	1 − *x*, 1 − *y*, 2 − *z*	N3—H3*A*⋯N5	2.15, 171
III(*a*)	1 + *x*, −1 + *y*, *z*	N1—H1*A*⋯N8	2.49, 155
		N1—H1*A*⋯C25	2.77, 130
		S1⋯C31	3.336 (1)
		S1⋯C26	3.430 (1)
III(*b*)	−1 + *x*, *y*, *z*	N5—H5*A*⋯N4	2.53, 159
		N5—H5*A*⋯C9	2.75, 140
		C10⋯S3	3.372 (1)
		C15⋯S3	3.311 (1)
		C22—H22⋯F4	2.44, 164
IV(*a*)	−1 + *x*, *y*, *z*	C4—H4⋯F4	2.46, 161
IV(*b*)	1 + *x*, *y*, *z*	C20—H20⋯F12	2.41, 161
V(*a*)	−*x*, 2 − *y*, 1 − *z*	F1⋯F10	2.907 (1), 137, 107
V(*b*)	*x*, −1 + *y*, *z*	F3⋯F10	2.923 (1), 115, 120
		C27—H27⋯C2	2.81, 129
V(*c*)	−*x*, 2 − *y*, 1 − *z*	F9⋯F9	2.845 (1), 127, 127
V(*d*)	1 − *x*, 1 − *y*, 1 − *z*	F2⋯F5	2.954 (1), 143, 119
V(*e*)	1 − *x*, 2 − *y*, 1 − *z*	F6⋯F7	2.946 (1), 142, 111
V(*f*)	−*x*, 2 − *y*, 1 − *z*	F11⋯F9	3.071 (1), 129, 97
VI	*x*, *y*, *z*	C5⋯O2	3.179 (1)
		C7⋯C14	3.308 (1)
VII	*x*, *y*, *z*	C3—H3⋯S3	2.84, 145
VIII	*x*, *y*, *z*	C17⋯S4	3.460 (1)
		C23⋯C30	3.295 (1)
IX	*x*, *y*, *z*	C12—H12⋯C18	2.82, 124
		C12—H12⋯C23	2.80, 133

The normalized values of hydrogen atoms given by *PARST* (Nardelli, 1995 ▸) were used for the hydrogen-bonding (Taylor & Kennard, 1983 ▸) analysis.

**Table 3:** Experimental details

	RZHCl	RZ
Crystal data		
Chemical formula	C_8_H_6_ClF_3_N_2_OS^+^·Cl^−^	C_8_H_5_F_3_N_2_OS
*M*_r_	270.66	234.20
Crystal system, space group	Monoclinic, *P*2_1_/*c*	Triclinic, *P* 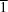
Temperature (K)	100	100
*a*, *b*, *c* (Å)	15.737 (8), 8.526 (4), 7.761 (4)	8.0824 (19), 11.788 (3), 19.745 (5)
α, β, γ (°)	90, 100.45 (2), 90	78.449 (9), 84.378 (8), 89.318 (9)
*V* (Å^3^)	1024.0 (9)	1834.2 (8)
*Z*	4	8
Radiation type	Mo *K*α	Mo *K*α
μ (mm^−1^)	0.60	0.37
Crystal size (mm)	0.39 × 0.08 × 0.05	0.20 × 0.20 × 0.03

Data collection		
Diffractometer	Bruker APEXII CCD	Bruker APEXII CCD
Absorption correction	Multi-scan (*SADABS*; Krause *et al.*, 2015 ▸)	Multi-scan (*SADABS*; Krause *et al.*, 2015 ▸)
*T*_min_, *T*_max_	0.572, 0.746	0.553, 0.746
No. of measured, independent and observed [*I* > 2σ(*I*)] reflections	5326, 2037, 1344	29801, 6730, 4593
*R*_int_	0.104	0.117

Refinement		
*R*[*F* ^2^ > 2σ(*F* ^2^)], *wR*(*F* ^2^), *S*	0.062, 0.157, 1.08	0.056, 0.130, 1.03
No. of reflections	2037	6730
No. of parameters	153	573
H-atom treatment	H atoms treated by a mixture of independent and constrained refinement	H atoms treated by a mixture of independent and constrained refinement
Δρ_max_, Δρ_min_ (e Å^−3^)	0.65, −0.60	0.51, −0.47

Computer programs: *APEX2* and *SAINT* (Bruker, 2009 ▸), *SHELXT2014/4* (Sheldrick, 2015*a* ▸), *SHELXL2016/6* (Sheldrick, 2015*b* ▸), *SHELXTL* (Sheldrick, 2008 ▸), *Mercury* (Macrae *et al.*, 2008 ▸) and *WinGX* (Farrugia, 2012 ▸).

## supplementary crystallographic information

### 2-Amino-6-(trifluoromethoxy)-1,3-benzothiazol-3-ium chloride (RZHCl) Crystal data

**Table:**

C_8_H_6_ClF_3_N_2_OS^+^·Cl^−^	*F*(000) = 544
*M**_r_* = 270.66	*D*_x_ = 1.756 Mg m^−^^3^
Monoclinic, *P*2_1_/*c*	Mo *K*α radiation, λ = 0.71073 Å
*a* = 15.737 (8) Å	Cell parameters from 2658 reflections
*b* = 8.526 (4) Å	θ = 2.7–29.8°
*c* = 7.761 (4) Å	µ = 0.60 mm^−^^1^
β = 100.45 (2)°	*T* = 100 K
*V* = 1024.0 (9) Å^3^	Plates, colorless
*Z* = 4	0.39 × 0.08 × 0.05 mm

### 2-Amino-6-(trifluoromethoxy)-1,3-benzothiazol-3-ium chloride (RZHCl) Data collection

**Table:**

Bruker APEXII CCD diffractometer	1344 reflections with *I* > 2σ(*I*)
φ and ω scans	*R*_int_ = 0.104
Absorption correction: multi-scan (SADABS; Krause *et al.*, 2015)	θ_max_ = 26.4°, θ_min_ = 2.6°
*T*_min_ = 0.572, *T*_max_ = 0.746	*h* = −19→19
5326 measured reflections	*k* = −10→10
2037 independent reflections	*l* = −9→9

### 2-Amino-6-(trifluoromethoxy)-1,3-benzothiazol-3-ium chloride (RZHCl) Refinement

**Table:**

Refinement on *F*^2^	0 restraints
Least-squares matrix: full	Hydrogen site location: mixed
*R*[*F*^2^ > 2σ(*F*^2^)] = 0.062	H atoms treated by a mixture of independent and constrained refinement
*wR*(*F*^2^) = 0.157	*w* = 1/[σ^2^(*F*_o_^2^) + (0.0525*P*)^2^ + 1.7896*P*] where *P* = (*F*_o_^2^ + 2*F*_c_^2^)/3
*S* = 1.08	(Δ/σ)_max_ < 0.001
2037 reflections	Δρ_max_ = 0.65 e Å^−^^3^
153 parameters	Δρ_min_ = −0.60 e Å^−^^3^

### 2-Amino-6-(trifluoromethoxy)-1,3-benzothiazol-3-ium chloride (RZHCl) Special details

**Table:**

Geometry. All esds (except the esd in the dihedral angle between two l.s. planes) are estimated using the full covariance matrix. The cell esds are taken into account individually in the estimation of esds in distances, angles and torsion angles; correlations between esds in cell parameters are only used when they are defined by crystal symmetry. An approximate (isotropic) treatment of cell esds is used for estimating esds involving l.s. planes.

### 2-Amino-6-(trifluoromethoxy)-1,3-benzothiazol-3-ium chloride (RZHCl) Fractional atomic coordinates and isotropic or equivalent isotropic displacement parameters (Å^2^)

**Table:**

	*x*	*y*	*z*	*U*_iso_*/*U*_eq_	
S1	0.09861 (8)	0.78340 (12)	0.44751 (16)	0.0149 (3)	
Cl1	0.12321 (8)	0.16795 (12)	0.39066 (17)	0.0221 (4)	
F1	0.4624 (2)	1.1822 (3)	0.8833 (5)	0.0363 (9)	
O1	0.3711 (2)	0.9930 (3)	0.8688 (4)	0.0181 (8)	
N2	0.1688 (2)	0.5156 (4)	0.5297 (5)	0.0127 (9)	
H2	0.1770	0.4136	0.5374	0.015*	
F2	0.3640 (3)	1.1687 (4)	0.6555 (5)	0.0637 (13)	
N1	0.0427 (3)	0.4997 (5)	0.3216 (6)	0.0207 (10)	
H1B	−0.004 (4)	0.546 (5)	0.254 (7)	0.020 (14)*	
H1A	0.051 (4)	0.396 (7)	0.319 (8)	0.040 (17)*	
F3	0.4660 (3)	1.0022 (4)	0.6932 (6)	0.0696 (15)	
C2	0.2266 (3)	0.6230 (5)	0.6236 (6)	0.0140 (10)	
C7	0.1975 (3)	0.7775 (5)	0.5902 (6)	0.0126 (10)	
C1	0.1005 (3)	0.5799 (5)	0.4278 (7)	0.0147 (11)	
C6	0.2447 (3)	0.9045 (5)	0.6695 (6)	0.0139 (11)	
H6	0.2253	1.0095	0.6492	0.017*	
C3	0.3029 (3)	0.5914 (5)	0.7342 (6)	0.0164 (11)	
H3	0.3222	0.4866	0.7567	0.020*	
C5	0.3213 (3)	0.8689 (5)	0.7791 (7)	0.0160 (11)	
C4	0.3515 (3)	0.7175 (5)	0.8127 (7)	0.0183 (11)	
H4	0.4050	0.6996	0.8889	0.022*	
C8	0.4151 (4)	1.0835 (6)	0.7748 (8)	0.0293 (14)	

### 2-Amino-6-(trifluoromethoxy)-1,3-benzothiazol-3-ium chloride (RZHCl) Atomic displacement parameters (Å^2^)

**Table:**

	*U*^11^	*U*^22^	*U*^33^	*U*^12^	*U*^13^	*U*^23^
S1	0.0197 (7)	0.0091 (5)	0.0146 (7)	0.0011 (5)	−0.0001 (5)	0.0011 (4)
Cl1	0.0307 (8)	0.0110 (5)	0.0215 (7)	0.0016 (5)	−0.0037 (6)	−0.0011 (5)
F1	0.037 (2)	0.0304 (16)	0.039 (2)	−0.0181 (14)	0.0001 (16)	−0.0117 (15)
O1	0.022 (2)	0.0206 (16)	0.011 (2)	−0.0081 (14)	0.0011 (15)	−0.0053 (13)
N2	0.017 (2)	0.0075 (16)	0.013 (2)	−0.0012 (15)	−0.0002 (17)	−0.0001 (15)
F2	0.069 (3)	0.053 (2)	0.057 (3)	−0.034 (2)	−0.019 (2)	0.032 (2)
N1	0.022 (3)	0.0119 (19)	0.025 (3)	−0.0015 (18)	−0.003 (2)	0.0025 (18)
F3	0.076 (3)	0.057 (2)	0.095 (4)	−0.039 (2)	0.067 (3)	−0.046 (2)
C2	0.023 (3)	0.010 (2)	0.009 (3)	0.0002 (18)	0.004 (2)	0.0002 (18)
C7	0.012 (3)	0.014 (2)	0.013 (3)	0.0005 (18)	0.005 (2)	0.0038 (18)
C1	0.020 (3)	0.008 (2)	0.017 (3)	−0.0040 (19)	0.007 (2)	0.0006 (18)
C6	0.023 (3)	0.011 (2)	0.009 (3)	0.0013 (18)	0.007 (2)	0.0002 (18)
C3	0.026 (3)	0.012 (2)	0.011 (3)	0.0027 (19)	0.003 (2)	0.0012 (18)
C5	0.025 (3)	0.015 (2)	0.011 (3)	−0.0073 (19)	0.011 (2)	−0.0031 (18)
C4	0.015 (3)	0.024 (2)	0.015 (3)	0.003 (2)	0.002 (2)	0.004 (2)
C8	0.034 (4)	0.024 (3)	0.030 (4)	−0.013 (2)	0.006 (3)	−0.007 (2)

### 2-Amino-6-(trifluoromethoxy)-1,3-benzothiazol-3-ium chloride (RZHCl) Geometric parameters (Å, º)

**Table:**

S1—C7	1.739 (5)	N1—H1A	0.89 (6)
S1—C1	1.742 (4)	F3—C8	1.306 (6)
F1—C8	1.321 (6)	C2—C3	1.371 (7)
O1—C8	1.338 (6)	C2—C7	1.403 (6)
O1—C5	1.422 (5)	C7—C6	1.392 (6)
N2—C1	1.332 (6)	C6—C5	1.376 (7)
N2—C2	1.398 (6)	C6—H6	0.9500
N2—H2	0.8800	C3—C4	1.393 (7)
F2—C8	1.328 (7)	C3—H3	0.9500
N1—C1	1.305 (6)	C5—C4	1.384 (6)
N1—H1B	0.91 (6)	C4—H4	0.9500
			
C7—S1—C1	90.0 (2)	C5—C6—H6	122.0
C8—O1—C5	117.1 (4)	C7—C6—H6	122.0
C1—N2—C2	114.7 (4)	C2—C3—C4	118.1 (4)
C1—N2—H2	122.6	C2—C3—H3	121.0
C2—N2—H2	122.6	C4—C3—H3	121.0
C1—N1—H1B	122 (3)	C6—C5—C4	123.7 (4)
C1—N1—H1A	116 (4)	C6—C5—O1	118.8 (4)
H1B—N1—H1A	121 (5)	C4—C5—O1	117.5 (5)
C3—C2—N2	127.6 (4)	C5—C4—C3	119.7 (5)
C3—C2—C7	121.3 (4)	C5—C4—H4	120.2
N2—C2—C7	111.0 (4)	C3—C4—H4	120.2
C6—C7—C2	121.2 (4)	F3—C8—F1	108.8 (5)
C6—C7—S1	127.1 (3)	F3—C8—F2	107.4 (5)
C2—C7—S1	111.7 (3)	F1—C8—F2	107.3 (4)
N1—C1—N2	123.7 (4)	F3—C8—O1	112.5 (4)
N1—C1—S1	123.8 (4)	F1—C8—O1	107.9 (5)
N2—C1—S1	112.5 (3)	F2—C8—O1	112.8 (5)
C5—C6—C7	116.1 (4)		

### 6-(Trifluoromethoxy)-1,3-benzothiazol-2-amine (RZ) Crystal data

**Table:**

C_8_H_5_F_3_N_2_OS	*Z* = 8
*M**_r_* = 234.20	*F*(000) = 944
Triclinic, *P*1	*D*_x_ = 1.696 Mg m^−^^3^
*a* = 8.0824 (19) Å	Mo *K*α radiation, λ = 0.71073 Å
*b* = 11.788 (3) Å	Cell parameters from 7465 reflections
*c* = 19.745 (5) Å	θ = 2.8–28.3°
α = 78.449 (9)°	µ = 0.37 mm^−^^1^
β = 84.378 (8)°	*T* = 100 K
γ = 89.318 (9)°	Plates, colorless
*V* = 1834.2 (8) Å^3^	0.20 × 0.20 × 0.03 mm

### 6-(Trifluoromethoxy)-1,3-benzothiazol-2-amine (RZ) Data collection

**Table:**

Bruker APEXII CCD diffractometer	4593 reflections with *I* > 2σ(*I*)
φ and ω scans	*R*_int_ = 0.117
Absorption correction: multi-scan (SADABS; Krause *et al.*, 2015)	θ_max_ = 25.5°, θ_min_ = 2.1°
*T*_min_ = 0.553, *T*_max_ = 0.746	*h* = −9→8
29801 measured reflections	*k* = −14→14
6730 independent reflections	*l* = −23→23

### 6-(Trifluoromethoxy)-1,3-benzothiazol-2-amine (RZ) Refinement

**Table:**

Refinement on *F*^2^	0 restraints
Least-squares matrix: full	Hydrogen site location: mixed
*R*[*F*^2^ > 2σ(*F*^2^)] = 0.056	H atoms treated by a mixture of independent and constrained refinement
*wR*(*F*^2^) = 0.130	*w* = 1/[σ^2^(*F*_o_^2^) + (0.0541*P*)^2^ + 0.7369*P*] where *P* = (*F*_o_^2^ + 2*F*_c_^2^)/3
*S* = 1.02	(Δ/σ)_max_ = 0.001
6730 reflections	Δρ_max_ = 0.51 e Å^−^^3^
573 parameters	Δρ_min_ = −0.47 e Å^−^^3^

### 6-(Trifluoromethoxy)-1,3-benzothiazol-2-amine (RZ) Special details

**Table:**

Geometry. All esds (except the esd in the dihedral angle between two l.s. planes) are estimated using the full covariance matrix. The cell esds are taken into account individually in the estimation of esds in distances, angles and torsion angles; correlations between esds in cell parameters are only used when they are defined by crystal symmetry. An approximate (isotropic) treatment of cell esds is used for estimating esds involving l.s. planes.

### 6-(Trifluoromethoxy)-1,3-benzothiazol-2-amine (RZ) Fractional atomic coordinates and isotropic or equivalent isotropic displacement parameters (Å^2^)

**Table:**

	*x*	*y*	*z*	*U*_iso_*/*U*_eq_	
S1	0.56739 (12)	0.27424 (7)	0.80617 (4)	0.0194 (2)	
S2	0.83832 (12)	0.45174 (7)	0.89575 (4)	0.0186 (2)	
S3	−0.00146 (12)	0.76611 (7)	0.82003 (4)	0.0193 (2)	
S4	−0.30623 (12)	0.95409 (7)	0.89386 (4)	0.0176 (2)	
F1	0.2905 (3)	0.5228 (2)	0.48186 (10)	0.0538 (8)	
F2	0.2508 (4)	0.3698 (2)	0.56134 (13)	0.0681 (9)	
F3	0.0949 (4)	0.5160 (3)	0.56353 (12)	0.0628 (8)	
F4	0.9015 (3)	0.7039 (2)	0.62797 (11)	0.0427 (6)	
F5	0.7172 (3)	0.7514 (2)	0.55647 (10)	0.0405 (6)	
F6	0.7410 (3)	0.84532 (19)	0.63754 (10)	0.0451 (7)	
F7	0.3242 (3)	0.9755 (2)	0.48390 (10)	0.0476 (7)	
F8	0.3079 (4)	0.8212 (2)	0.56344 (11)	0.0504 (8)	
F9	0.0973 (4)	0.9316 (2)	0.54890 (11)	0.0527 (7)	
F10	−0.1373 (3)	1.3300 (2)	0.63105 (11)	0.0463 (7)	
F11	−0.0685 (3)	1.2372 (2)	0.54945 (10)	0.0422 (7)	
F12	−0.2758 (3)	1.1814 (2)	0.62482 (11)	0.0538 (8)	
O1	0.3591 (3)	0.5281 (2)	0.58387 (11)	0.0280 (6)	
O2	0.6333 (3)	0.6668 (2)	0.66125 (11)	0.0231 (6)	
O3	0.3167 (4)	0.9962 (2)	0.58944 (12)	0.0331 (7)	
O4	−0.0152 (3)	1.1590 (2)	0.65325 (11)	0.0253 (6)	
N1	0.4995 (5)	0.2583 (3)	0.94561 (15)	0.0191 (7)	
H1A	0.599 (5)	0.237 (3)	0.9497 (16)	0.017 (10)*	
H1B	0.463 (5)	0.292 (4)	0.980 (2)	0.042 (13)*	
N2	0.3463 (4)	0.3895 (2)	0.86966 (13)	0.0167 (6)	
N3	0.7894 (4)	0.4741 (3)	1.02897 (15)	0.0215 (7)	
H3A	0.847 (5)	0.414 (3)	1.0362 (18)	0.030 (12)*	
H3B	0.743 (5)	0.504 (3)	1.0596 (19)	0.029 (12)*	
N4	0.6520 (4)	0.6036 (2)	0.94687 (13)	0.0179 (7)	
N5	0.0104 (5)	0.7614 (3)	0.95743 (15)	0.0185 (7)	
H5A	−0.090 (6)	0.728 (3)	0.9646 (18)	0.033 (12)*	
H5B	0.041 (7)	0.795 (4)	0.987 (2)	0.068 (18)*	
N6	0.1865 (4)	0.8916 (2)	0.87642 (13)	0.0175 (7)	
N7	−0.2997 (4)	0.9781 (3)	1.02592 (15)	0.0218 (7)	
H7A	−0.362 (5)	0.915 (3)	1.0386 (16)	0.016 (9)*	
H7B	−0.266 (5)	1.009 (3)	1.0575 (18)	0.027 (11)*	
N8	−0.1349 (4)	1.1049 (2)	0.93992 (13)	0.0171 (6)	
C1	0.4623 (5)	0.3124 (3)	0.88061 (16)	0.0185 (8)	
C2	0.3336 (5)	0.4262 (3)	0.79884 (16)	0.0172 (8)	
C12	0.5408 (5)	0.7238 (3)	0.76878 (16)	0.0192 (8)	
H12	0.475160	0.782215	0.744004	0.023*	
C23	0.1382 (4)	0.8618 (3)	0.76540 (16)	0.0160 (8)	
C20	0.3703 (5)	1.0258 (3)	0.69917 (17)	0.0230 (8)	
H20	0.450304	1.081977	0.675480	0.028*	
C14	0.7326 (5)	0.5640 (3)	0.76693 (16)	0.0192 (8)	
H14	0.796259	0.514646	0.742027	0.023*	
C19	0.3441 (5)	1.0046 (3)	0.77075 (17)	0.0205 (8)	
H19	0.405089	1.046423	0.796581	0.025*	
C9	0.7506 (4)	0.5159 (3)	0.96437 (16)	0.0160 (8)	
C30	−0.1529 (5)	1.0603 (3)	0.76231 (16)	0.0175 (8)	
H30	−0.207468	1.010101	0.739374	0.021*	
C31	−0.1760 (4)	1.0495 (3)	0.83368 (16)	0.0153 (7)	
C25	−0.2392 (4)	1.0181 (3)	0.96022 (16)	0.0156 (7)	
C26	−0.0953 (4)	1.1236 (3)	0.86839 (15)	0.0146 (7)	
C11	0.5392 (5)	0.7088 (3)	0.84009 (16)	0.0196 (8)	
H11	0.471794	0.756926	0.864594	0.023*	
C10	0.6358 (4)	0.6237 (3)	0.87584 (16)	0.0160 (8)	
C17	0.0717 (5)	0.8133 (3)	0.89124 (16)	0.0173 (8)	
C22	0.1616 (5)	0.8820 (3)	0.69313 (16)	0.0209 (8)	
H22	0.099841	0.841139	0.667017	0.025*	
C6	0.4514 (5)	0.4040 (3)	0.68358 (17)	0.0206 (8)	
H6	0.526557	0.368110	0.654413	0.025*	
C15	0.7294 (4)	0.5499 (3)	0.83875 (16)	0.0164 (8)	
C32	−0.1223 (5)	1.2251 (4)	0.61556 (18)	0.0308 (10)	
C7	0.4453 (5)	0.3729 (3)	0.75555 (16)	0.0174 (8)	
C28	0.0382 (5)	1.2201 (3)	0.75845 (16)	0.0208 (8)	
H28	0.112366	1.277598	0.731590	0.025*	
C18	0.2277 (4)	0.9216 (3)	0.80461 (16)	0.0158 (8)	
C16	0.7463 (5)	0.7402 (3)	0.62168 (17)	0.0275 (9)	
C5	0.3445 (5)	0.4888 (3)	0.65630 (16)	0.0202 (8)	
C13	0.6395 (5)	0.6524 (3)	0.73365 (16)	0.0183 (8)	
C3	0.2279 (5)	0.5104 (3)	0.76902 (17)	0.0204 (8)	
H3	0.151349	0.546462	0.797609	0.025*	
C29	−0.0478 (5)	1.1467 (3)	0.72619 (16)	0.0195 (8)	
C21	0.2789 (5)	0.9644 (3)	0.66162 (16)	0.0216 (8)	
C27	0.0141 (5)	1.2081 (3)	0.82977 (16)	0.0199 (8)	
H27	0.071741	1.257234	0.852371	0.024*	
C4	0.2335 (5)	0.5424 (3)	0.69743 (17)	0.0239 (9)	
H4	0.161571	0.600681	0.676698	0.029*	
C8	0.2511 (6)	0.4824 (4)	0.54838 (19)	0.0387 (11)	
C24	0.2624 (6)	0.9305 (4)	0.54759 (18)	0.0347 (11)	

### 6-(Trifluoromethoxy)-1,3-benzothiazol-2-amine (RZ) Atomic displacement parameters (Å^2^)

**Table:**

	*U*^11^	*U*^22^	*U*^33^	*U*^12^	*U*^13^	*U*^23^
S1	0.0208 (6)	0.0135 (5)	0.0253 (4)	0.0032 (4)	−0.0030 (4)	−0.0070 (3)
S2	0.0197 (6)	0.0137 (5)	0.0232 (4)	0.0015 (4)	−0.0018 (4)	−0.0061 (3)
S3	0.0192 (6)	0.0156 (5)	0.0245 (4)	−0.0047 (4)	−0.0002 (4)	−0.0084 (3)
S4	0.0187 (6)	0.0141 (4)	0.0211 (4)	−0.0028 (4)	−0.0018 (3)	−0.0062 (3)
F1	0.054 (2)	0.084 (2)	0.0219 (12)	0.0105 (15)	−0.0067 (11)	−0.0060 (12)
F2	0.123 (3)	0.0444 (18)	0.0446 (15)	−0.0135 (17)	−0.0252 (16)	−0.0176 (13)
F3	0.0352 (19)	0.114 (3)	0.0434 (15)	0.0000 (17)	−0.0094 (12)	−0.0228 (15)
F4	0.0258 (16)	0.0544 (16)	0.0435 (13)	−0.0024 (13)	0.0007 (11)	−0.0014 (11)
F5	0.0532 (19)	0.0484 (15)	0.0201 (11)	−0.0052 (13)	−0.0036 (10)	−0.0066 (10)
F6	0.079 (2)	0.0220 (13)	0.0322 (12)	−0.0116 (12)	0.0033 (12)	−0.0042 (10)
F7	0.066 (2)	0.0562 (17)	0.0188 (11)	−0.0080 (14)	0.0006 (11)	−0.0043 (10)
F8	0.081 (2)	0.0370 (15)	0.0331 (13)	0.0120 (14)	0.0028 (12)	−0.0117 (11)
F9	0.042 (2)	0.080 (2)	0.0350 (13)	−0.0092 (15)	−0.0155 (11)	−0.0022 (12)
F10	0.067 (2)	0.0360 (15)	0.0338 (13)	0.0146 (13)	−0.0077 (12)	−0.0008 (11)
F11	0.0503 (18)	0.0570 (16)	0.0177 (11)	−0.0113 (13)	−0.0014 (10)	−0.0041 (10)
F12	0.0317 (18)	0.087 (2)	0.0378 (13)	−0.0198 (15)	−0.0083 (11)	0.0024 (13)
O1	0.0260 (18)	0.0327 (16)	0.0232 (13)	−0.0004 (12)	−0.0024 (11)	−0.0001 (11)
O2	0.0265 (17)	0.0262 (14)	0.0180 (12)	−0.0072 (12)	−0.0043 (10)	−0.0059 (10)
O3	0.043 (2)	0.0345 (16)	0.0208 (13)	−0.0110 (14)	0.0025 (12)	−0.0043 (11)
O4	0.0307 (18)	0.0277 (15)	0.0175 (12)	0.0016 (12)	−0.0001 (11)	−0.0053 (10)
N1	0.017 (2)	0.0176 (17)	0.0231 (16)	0.0009 (14)	−0.0057 (13)	−0.0040 (13)
N2	0.0180 (19)	0.0115 (15)	0.0211 (14)	−0.0006 (13)	−0.0026 (12)	−0.0044 (11)
N3	0.025 (2)	0.0198 (18)	0.0203 (17)	0.0073 (15)	−0.0038 (14)	−0.0047 (14)
N4	0.0171 (19)	0.0159 (16)	0.0211 (14)	−0.0022 (13)	−0.0013 (12)	−0.0049 (11)
N5	0.020 (2)	0.0126 (16)	0.0225 (16)	−0.0034 (14)	0.0000 (13)	−0.0035 (12)
N6	0.0194 (19)	0.0119 (15)	0.0216 (15)	0.0002 (13)	−0.0030 (12)	−0.0035 (11)
N7	0.025 (2)	0.0221 (18)	0.0192 (16)	−0.0118 (15)	−0.0006 (13)	−0.0063 (14)
N8	0.0158 (18)	0.0155 (15)	0.0206 (14)	−0.0016 (13)	−0.0003 (12)	−0.0058 (11)
C1	0.022 (2)	0.0091 (17)	0.0248 (18)	−0.0068 (16)	0.0001 (15)	−0.0051 (14)
C2	0.020 (2)	0.0097 (17)	0.0231 (17)	−0.0044 (15)	−0.0002 (14)	−0.0057 (13)
C12	0.016 (2)	0.0176 (19)	0.0242 (18)	−0.0021 (15)	−0.0072 (14)	−0.0014 (14)
C23	0.013 (2)	0.0126 (17)	0.0223 (17)	−0.0006 (14)	−0.0010 (14)	−0.0045 (14)
C20	0.023 (2)	0.0146 (19)	0.031 (2)	−0.0085 (16)	0.0002 (16)	−0.0028 (15)
C14	0.022 (2)	0.0131 (18)	0.0241 (18)	−0.0049 (15)	−0.0008 (15)	−0.0086 (14)
C19	0.019 (2)	0.0169 (19)	0.0269 (18)	−0.0057 (16)	−0.0052 (15)	−0.0067 (15)
C9	0.011 (2)	0.0144 (18)	0.0234 (18)	−0.0043 (15)	0.0009 (14)	−0.0076 (14)
C30	0.017 (2)	0.0162 (18)	0.0224 (18)	0.0032 (15)	−0.0052 (14)	−0.0096 (14)
C31	0.016 (2)	0.0071 (16)	0.0226 (17)	0.0007 (14)	−0.0020 (14)	−0.0029 (13)
C25	0.011 (2)	0.0148 (18)	0.0234 (18)	0.0001 (15)	−0.0042 (14)	−0.0087 (14)
C26	0.013 (2)	0.0110 (17)	0.0211 (17)	0.0046 (14)	−0.0037 (14)	−0.0048 (13)
C11	0.018 (2)	0.0162 (19)	0.0263 (18)	−0.0031 (16)	−0.0001 (15)	−0.0090 (14)
C10	0.017 (2)	0.0096 (17)	0.0207 (17)	−0.0047 (15)	0.0005 (14)	−0.0032 (13)
C17	0.018 (2)	0.0110 (18)	0.0242 (18)	0.0026 (15)	−0.0032 (14)	−0.0055 (14)
C22	0.021 (2)	0.021 (2)	0.0221 (18)	0.0004 (16)	−0.0032 (15)	−0.0083 (15)
C6	0.020 (2)	0.0185 (19)	0.0258 (18)	−0.0041 (16)	−0.0014 (15)	−0.0100 (15)
C15	0.014 (2)	0.0098 (17)	0.0252 (18)	−0.0031 (14)	−0.0018 (14)	−0.0033 (13)
C32	0.031 (3)	0.038 (3)	0.022 (2)	−0.006 (2)	−0.0008 (17)	−0.0034 (17)
C7	0.014 (2)	0.0132 (18)	0.0260 (18)	−0.0011 (15)	−0.0028 (14)	−0.0069 (14)
C28	0.016 (2)	0.020 (2)	0.0247 (18)	−0.0007 (16)	0.0038 (14)	−0.0022 (15)
C18	0.015 (2)	0.0106 (17)	0.0230 (17)	0.0040 (15)	−0.0040 (14)	−0.0054 (13)
C16	0.035 (3)	0.025 (2)	0.0230 (19)	0.0025 (19)	−0.0042 (16)	−0.0054 (16)
C5	0.018 (2)	0.021 (2)	0.0207 (17)	−0.0041 (16)	−0.0023 (14)	−0.0020 (14)
C13	0.018 (2)	0.0164 (18)	0.0205 (17)	−0.0068 (15)	−0.0032 (14)	−0.0029 (14)
C3	0.018 (2)	0.0160 (19)	0.0272 (18)	−0.0005 (16)	0.0005 (15)	−0.0062 (15)
C29	0.022 (2)	0.0186 (19)	0.0182 (17)	0.0042 (16)	0.0004 (14)	−0.0060 (14)
C21	0.020 (2)	0.022 (2)	0.0218 (18)	−0.0005 (16)	0.0002 (15)	−0.0027 (14)
C27	0.023 (2)	0.0149 (18)	0.0236 (18)	0.0006 (16)	−0.0042 (15)	−0.0062 (14)
C4	0.022 (2)	0.0169 (19)	0.033 (2)	0.0006 (16)	−0.0065 (16)	−0.0024 (15)
C8	0.042 (3)	0.052 (3)	0.022 (2)	0.001 (2)	−0.0040 (18)	−0.0069 (19)
C24	0.044 (3)	0.038 (3)	0.021 (2)	0.000 (2)	−0.0057 (18)	−0.0024 (17)

### 6-(Trifluoromethoxy)-1,3-benzothiazol-2-amine (RZ) Geometric parameters (Å, º)

**Table:**

S1—C7	1.739 (4)	N6—C18	1.399 (4)
S1—C1	1.759 (3)	N7—C25	1.335 (4)
S2—C15	1.740 (3)	N7—H7A	0.88 (4)
S2—C9	1.766 (3)	N7—H7B	0.85 (4)
S3—C23	1.736 (3)	N8—C25	1.307 (4)
S3—C17	1.766 (3)	N8—C26	1.391 (4)
S4—C31	1.742 (3)	C2—C3	1.381 (5)
S4—C25	1.768 (3)	C2—C7	1.411 (4)
F1—C8	1.313 (4)	C12—C11	1.383 (4)
F2—C8	1.300 (5)	C12—C13	1.391 (5)
F3—C8	1.342 (5)	C12—H12	0.9500
F4—C16	1.330 (5)	C23—C22	1.393 (4)
F5—C16	1.312 (4)	C23—C18	1.400 (5)
F6—C16	1.337 (4)	C20—C19	1.381 (4)
F7—C24	1.315 (4)	C20—C21	1.398 (5)
F8—C24	1.320 (5)	C20—H20	0.9500
F9—C24	1.332 (5)	C14—C13	1.374 (5)
F10—C32	1.334 (4)	C14—C15	1.392 (4)
F11—C32	1.315 (4)	C14—H14	0.9500
F12—C32	1.331 (5)	C19—C18	1.387 (5)
O1—C8	1.351 (5)	C19—H19	0.9500
O1—C5	1.406 (4)	C30—C29	1.371 (5)
O2—C16	1.342 (5)	C30—C31	1.383 (4)
O2—C13	1.411 (4)	C30—H30	0.9500
O3—C24	1.345 (5)	C31—C26	1.413 (5)
O3—C21	1.403 (4)	C26—C27	1.393 (5)
O4—C32	1.337 (5)	C11—C10	1.386 (5)
O4—C29	1.417 (4)	C11—H11	0.9500
N1—C1	1.374 (4)	C10—C15	1.414 (4)
N1—H1A	0.84 (4)	C22—C21	1.376 (5)
N1—H1B	0.88 (4)	C22—H22	0.9500
N2—C1	1.301 (4)	C6—C5	1.375 (5)
N2—C2	1.392 (4)	C6—C7	1.391 (5)
N3—C9	1.338 (4)	C6—H6	0.9500
N3—H3A	0.84 (4)	C28—C27	1.381 (4)
N3—H3B	0.82 (4)	C28—C29	1.400 (5)
N4—C9	1.309 (4)	C28—H28	0.9500
N4—C10	1.394 (4)	C5—C4	1.383 (5)
N5—C17	1.374 (4)	C3—C4	1.384 (5)
N5—H5A	0.89 (5)	C3—H3	0.9500
N5—H5B	0.82 (5)	C27—H27	0.9500
N6—C17	1.288 (4)	C4—H4	0.9500
			
C7—S1—C1	88.72 (16)	N6—C17—N5	124.8 (3)
C15—S2—C9	88.86 (16)	N6—C17—S3	116.1 (2)
C23—S3—C17	88.43 (16)	N5—C17—S3	119.0 (3)
C31—S4—C25	88.76 (16)	C21—C22—C23	116.4 (3)
C8—O1—C5	116.5 (3)	C21—C22—H22	121.8
C16—O2—C13	115.7 (3)	C23—C22—H22	121.8
C24—O3—C21	120.0 (3)	C5—C6—C7	117.4 (3)
C32—O4—C29	115.5 (3)	C5—C6—H6	121.3
C1—N1—H1A	117 (2)	C7—C6—H6	121.3
C1—N1—H1B	116 (3)	C14—C15—C10	121.6 (3)
H1A—N1—H1B	110 (4)	C14—C15—S2	128.8 (3)
C1—N2—C2	110.8 (3)	C10—C15—S2	109.6 (2)
C9—N3—H3A	119 (2)	F11—C32—F12	108.3 (3)
C9—N3—H3B	116 (3)	F11—C32—F10	108.5 (3)
H3A—N3—H3B	124 (4)	F12—C32—F10	105.5 (3)
C9—N4—C10	110.5 (3)	F11—C32—O4	108.7 (3)
C17—N5—H5A	120 (2)	F12—C32—O4	113.5 (3)
C17—N5—H5B	112 (4)	F10—C32—O4	112.2 (3)
H5A—N5—H5B	119 (4)	C6—C7—C2	121.2 (3)
C17—N6—C18	110.7 (3)	C6—C7—S1	129.0 (3)
C25—N7—H7A	123 (2)	C2—C7—S1	109.7 (2)
C25—N7—H7B	118 (3)	C27—C28—C29	119.2 (3)
H7A—N7—H7B	118 (3)	C27—C28—H28	120.4
C25—N8—C26	110.3 (3)	C29—C28—H28	120.4
N2—C1—N1	123.7 (3)	C19—C18—N6	125.9 (3)
N2—C1—S1	116.0 (2)	C19—C18—C23	119.3 (3)
N1—C1—S1	120.2 (3)	N6—C18—C23	114.8 (3)
C3—C2—N2	126.0 (3)	F5—C16—F4	108.6 (3)
C3—C2—C7	119.2 (3)	F5—C16—F6	108.1 (3)
N2—C2—C7	114.7 (3)	F4—C16—F6	105.8 (3)
C11—C12—C13	119.4 (3)	F5—C16—O2	108.7 (3)
C11—C12—H12	120.3	F4—C16—O2	112.8 (3)
C13—C12—H12	120.3	F6—C16—O2	112.7 (3)
C22—C23—C18	122.5 (3)	C6—C5—C4	122.6 (3)
C22—C23—S3	127.6 (3)	C6—C5—O1	118.0 (3)
C18—C23—S3	109.9 (2)	C4—C5—O1	119.2 (3)
C19—C20—C21	119.9 (3)	C14—C13—C12	122.9 (3)
C19—C20—H20	120.1	C14—C13—O2	119.0 (3)
C21—C20—H20	120.1	C12—C13—O2	117.9 (3)
C13—C14—C15	117.0 (3)	C2—C3—C4	119.9 (3)
C13—C14—H14	121.5	C2—C3—H3	120.0
C15—C14—H14	121.5	C4—C3—H3	120.0
C20—C19—C18	119.4 (3)	C30—C29—C28	123.0 (3)
C20—C19—H19	120.3	C30—C29—O4	119.3 (3)
C18—C19—H19	120.3	C28—C29—O4	117.6 (3)
N4—C9—N3	124.9 (3)	C22—C21—C20	122.6 (3)
N4—C9—S2	115.9 (2)	C22—C21—O3	123.8 (3)
N3—C9—S2	119.2 (3)	C20—C21—O3	113.6 (3)
C29—C30—C31	117.0 (3)	C28—C27—C26	119.7 (3)
C29—C30—H30	121.5	C28—C27—H27	120.2
C31—C30—H30	121.5	C26—C27—H27	120.2
C30—C31—C26	122.0 (3)	C5—C4—C3	119.6 (3)
C30—C31—S4	128.6 (3)	C5—C4—H4	120.2
C26—C31—S4	109.3 (2)	C3—C4—H4	120.2
N8—C25—N7	124.8 (3)	F2—C8—F1	110.3 (3)
N8—C25—S4	116.0 (2)	F2—C8—F3	107.3 (4)
N7—C25—S4	119.2 (3)	F1—C8—F3	107.0 (3)
N8—C26—C27	125.4 (3)	F2—C8—O1	113.1 (4)
N8—C26—C31	115.6 (3)	F1—C8—O1	107.9 (4)
C27—C26—C31	119.0 (3)	F3—C8—O1	111.2 (3)
C12—C11—C10	120.0 (3)	F7—C24—F8	109.2 (3)
C12—C11—H11	120.0	F7—C24—F9	107.8 (3)
C10—C11—H11	120.0	F8—C24—F9	107.4 (4)
C11—C10—N4	125.7 (3)	F7—C24—O3	107.2 (4)
C11—C10—C15	119.0 (3)	F8—C24—O3	113.3 (3)
N4—C10—C15	115.2 (3)	F9—C24—O3	111.9 (3)
			
C2—N2—C1—N1	−178.1 (3)	N2—C2—C7—C6	−177.6 (3)
C2—N2—C1—S1	−0.9 (4)	C3—C2—C7—S1	178.1 (3)
C7—S1—C1—N2	0.8 (3)	N2—C2—C7—S1	0.2 (4)
C7—S1—C1—N1	178.1 (3)	C1—S1—C7—C6	177.0 (3)
C1—N2—C2—C3	−177.3 (4)	C1—S1—C7—C2	−0.6 (3)
C1—N2—C2—C7	0.4 (4)	C20—C19—C18—N6	−179.3 (3)
C17—S3—C23—C22	−179.0 (3)	C20—C19—C18—C23	−0.6 (5)
C17—S3—C23—C18	−0.2 (3)	C17—N6—C18—C19	178.7 (3)
C21—C20—C19—C18	0.5 (5)	C17—N6—C18—C23	−0.1 (4)
C10—N4—C9—N3	−179.1 (3)	C22—C23—C18—C19	0.2 (5)
C10—N4—C9—S2	1.7 (4)	S3—C23—C18—C19	−178.6 (3)
C15—S2—C9—N4	−1.2 (3)	C22—C23—C18—N6	179.0 (3)
C15—S2—C9—N3	179.6 (3)	S3—C23—C18—N6	0.2 (4)
C29—C30—C31—C26	−0.1 (5)	C13—O2—C16—F5	−175.2 (3)
C29—C30—C31—S4	177.0 (3)	C13—O2—C16—F4	64.3 (4)
C25—S4—C31—C30	−178.7 (3)	C13—O2—C16—F6	−55.4 (4)
C25—S4—C31—C26	−1.3 (3)	C7—C6—C5—C4	−0.2 (6)
C26—N8—C25—N7	179.3 (3)	C7—C6—C5—O1	174.7 (3)
C26—N8—C25—S4	−2.5 (4)	C8—O1—C5—C6	98.8 (4)
C31—S4—C25—N8	2.3 (3)	C8—O1—C5—C4	−86.2 (4)
C31—S4—C25—N7	−179.4 (3)	C15—C14—C13—C12	−2.1 (5)
C25—N8—C26—C27	−179.1 (3)	C15—C14—C13—O2	−177.3 (3)
C25—N8—C26—C31	1.4 (4)	C11—C12—C13—C14	2.3 (5)
C30—C31—C26—N8	177.9 (3)	C11—C12—C13—O2	177.5 (3)
S4—C31—C26—N8	0.2 (4)	C16—O2—C13—C14	−92.6 (4)
C30—C31—C26—C27	−1.7 (5)	C16—O2—C13—C12	92.0 (4)
S4—C31—C26—C27	−179.3 (3)	N2—C2—C3—C4	177.0 (3)
C13—C12—C11—C10	0.3 (5)	C7—C2—C3—C4	−0.6 (5)
C12—C11—C10—N4	176.5 (3)	C31—C30—C29—C28	1.8 (5)
C12—C11—C10—C15	−2.7 (5)	C31—C30—C29—O4	177.8 (3)
C9—N4—C10—C11	179.1 (3)	C27—C28—C29—C30	−1.8 (5)
C9—N4—C10—C15	−1.6 (4)	C27—C28—C29—O4	−177.8 (3)
C18—N6—C17—N5	176.4 (3)	C32—O4—C29—C30	87.5 (4)
C18—N6—C17—S3	−0.1 (4)	C32—O4—C29—C28	−96.3 (4)
C23—S3—C17—N6	0.2 (3)	C23—C22—C21—C20	−0.3 (5)
C23—S3—C17—N5	−176.5 (3)	C23—C22—C21—O3	−179.3 (3)
C18—C23—C22—C21	0.3 (5)	C19—C20—C21—C22	0.0 (6)
S3—C23—C22—C21	178.8 (3)	C19—C20—C21—O3	179.0 (3)
C13—C14—C15—C10	−0.5 (5)	C24—O3—C21—C22	−13.4 (6)
C13—C14—C15—S2	−177.1 (3)	C24—O3—C21—C20	167.6 (4)
C11—C10—C15—C14	2.9 (5)	C29—C28—C27—C26	−0.1 (5)
N4—C10—C15—C14	−176.5 (3)	N8—C26—C27—C28	−177.7 (3)
C11—C10—C15—S2	−179.9 (3)	C31—C26—C27—C28	1.7 (5)
N4—C10—C15—S2	0.7 (4)	C6—C5—C4—C3	−0.1 (6)
C9—S2—C15—C14	177.1 (3)	O1—C5—C4—C3	−174.9 (3)
C9—S2—C15—C10	0.2 (3)	C2—C3—C4—C5	0.5 (5)
C29—O4—C32—F11	174.4 (3)	C5—O1—C8—F2	−53.5 (5)
C29—O4—C32—F12	−65.1 (4)	C5—O1—C8—F1	−175.7 (3)
C29—O4—C32—F10	54.4 (4)	C5—O1—C8—F3	67.2 (4)
C5—C6—C7—C2	0.1 (5)	C21—O3—C24—F7	−175.9 (3)
C5—C6—C7—S1	−177.3 (3)	C21—O3—C24—F8	−55.4 (5)
C3—C2—C7—C6	0.3 (5)	C21—O3—C24—F9	66.1 (4)
